# Tau Imaging: Use and Implementation in New Diagnostic and Therapeutic Paradigms for Alzheimer’s Disease

**DOI:** 10.3390/geriatrics10010027

**Published:** 2025-02-14

**Authors:** Alexandra Gogola, Brian J. Lopresti, Davneet S. Minhas, Oscar Lopez, Ann Cohen, Victor L. Villemagne

**Affiliations:** 1 Department of Radiology, School of Medicine, University of Pittsburgh, Pittsburgh, PA 15213, USA; brianl@pitt.edu (B.J.L.); dam148@pitt.edu (D.S.M.); 2 Department of Neurology, School of Medicine, University of Pittsburgh, Pittsburgh, PA 15213, USA; ollopez@pitt.edu; 3 Department of Psychiatry, School of Medicine, University of Pittsburgh, Pittsburgh, PA 15213, USA; anc2597@pitt.edu (A.C.); victor.villemagne@pitt.edu (V.L.V.)

**Keywords:** tau PET, Alzheimer’s disease, cognitive decline

## Abstract

Alzheimer’s disease (AD) affects an estimated 6.9 million older adults in the United States and is projected to impact as many as 13.8 million people by 2060. As studies continue to search for ways to combat the development and progression of AD, it is imperative to ensure that confident diagnoses can be made before the onset of severe clinical symptoms and new therapies can be evaluated effectively. Tau positron emission tomography (PET) has emerged as one method that may be capable of both, given its ability to recognize the presence of tau, a primary pathologic hallmark of AD; its usefulness in determining the spatial distribution of tau, which is necessary for differentiating AD from other tauopathies; and its association with measures of cognition. This review aims to evaluate the scope of tau PET’s utility in clinical trials and practice. Firstly, the potential of using tau PET for differential diagnoses, distinguishing AD from other dementias, is considered. Next, the value of tau PET as a tool for staging disease progression is investigated. Finally, tau PET as a prognostic method for identifying the individuals most at risk of cognitive decline and, therefore, most in need of, and likely to benefit from, intervention, is discussed.

## 1. Introduction

Alzheimer’s disease (AD) dementia globally affects approximately 32 million older adults, and an estimated 22% of adults over 50 years of age have preclinical AD, prodromal AD, or AD dementia [1]. As the search for methods to combat the development and progression of AD continues, it is imperative that confident early diagnoses can be made and new therapies can be effectively evaluated before the presence of irreversible neurologic deficits. This necessitates efficient biomarker use for the early detection of AD, limiting long-term costs and acting as accurate surrogates for AD-related cognitive decline.

Much genetic and biochemical evidence exists to support the view that amyloid-β (Aβ) is the antecedent and the potential initiator of a pathologic cascade that includes tau deposition, neurodegeneration, and ultimately dementia [2,3]. It would then stand to reason that Aβ is the ideal biomarker for indicating early AD. However, a growing body of evidence suggests that this simple model of AD pathophysiology is neither universal nor complete. The assessment of Aβ can identify individuals in the preclinical phase of the disease, when 25–35% of cognitively unimpaired older adults present elevated Aβ deposits [4,5,6]. On the other hand, Aβ load is poorly associated with cognitive impairment and decline in AD, and the associations that have been found are mediated by the presence of tau [7,8]. These findings suggest that while Aβ is necessary for AD, as it is presently defined, to develop and occurs early in the disease’s progression, it alone is not sufficient to cause AD-related cognitive decline [9] and, therefore, is likely not an exclusive biomarker for evaluating AD in clinical settings. Tau PET is required to obtain a comprehensive picture and the stage of the underlying pathological process. All tau tracers have been shown to follow the hierarchical regional distribution of tau deposits in the brain, described by the neuropathological Braak and Delacourte staging [10,11] showing tau deposits predominantly in the isocortical pyramidal layer [12]. Some have proposed a simpler paradigm using tau PET as a singular method to assess AD and aid differential diagnoses from other dementing neurodegenerative conditions [13]. The evidence suggests that the effective use of tau PET can differentially diagnose AD [14,15], forecast neurodegeneration [16,17,18], and predict future cognitive decline [19,20,21], all of which position it as the optimal complementary tool for the study and staging of AD.

This review surveys the utility of tau PET as a tool for staging disease progression, supporting differential diagnoses, and determining prognoses determination and projects the scope of tau PET’s potential utility in clinical trials and practice as progress is made toward effective therapeutic interventions for AD.

## 2. Tau PET in Differential Diagnosis

AD may be the most common neurodegenerative disease defined by the presence of tau pathology, but it is not the only such condition characterized by tau aggregation. The tau pathology characteristic of AD is comprised of a mixture of all 3R and 4R tau species (3R/4R), as these are the tau aggregates that define chronic traumatic encephalopathy (CTE), and the majority of PET tracers developed thus far target the aggregates of 3R/4R tau [22]. While plasma tau isoforms, especially p-tau217 [23], are better predictors of Aβ than of tau, they are not yet as good at predicting imminent cognitive decline as the cortical tau assessed by PET is [20,24]. Moreover, fluid tau biomarkers cannot display the regional distribution of tau deposits in the brain that are intimately related to the clinical phenotype [24].

Primary tauopathies are a class of disorders where tau is the sole defining pathology, but they are distinct from the mixed 3R/4R tau pathology characteristic of AD. For example, the tau pathology found in corticobasal degeneration (CBD) and progressive supranuclear palsy (PSP) is predominantly comprised of 4R species, while those in Pick’s disease are mainly 3R [25,26,27,28]. Therefore, if tau PET is to potentially act as a singular biomarker for evaluating AD, it must be able to differentiate AD from other tauopathies, distinguishing which tau species are aggregated. All known tau tracers used in human studies to date are specific to 3R/4R tau aggregates and do not indicate other tau species in vivo. There are some tau tracers, namely PI2620 and PM-PBB3, that also display an affinity for 4R tau, albeit at a much lower level than for the 3R/4R tau in AD. For that purpose, there has been a concerted effort to develop PET tracers that are sensitive to and ultimately specific to 4R tauopathies, an effort that is complicated by the fact that there is a significantly much lower density of 4R tau pathology in PSP or CBD than the one observed with 3R/4R tau deposits in AD [29].

Previous studies have shown that tau imaging is highly specific and is exceptionally accurate for differential diagnoses against other neurodegenerative conditions at the advanced stages of AD dementia, although it is much less accurate at the prodromal (MCI) stage of AD [14,30]. Several studies have shown that the differential diagnosis of AD can be achieved with a variety of tau PET radiotracers [13,14,30,31,32].

Work with [^18^F]flortaucipir (FTP) has been successful in differentiating AD from a variety of other disorders. One study found that the cross-sectional evaluation of the ratio of FTP SUVR in the midbrain to the inferior temporal region can differentiate high probability AD neuropathologic change (ADNC) from low probability ADNC and FTLD with areas under the receiver operating curve (AUROCs) of 0.94 or greater [31]. Another achieved the differentiation of AD from FTLD using the data-driven, scaled subprofile model (SSM)/principal component analysis (PCA)-derived ratio of gray matter to white matter FTP uptake, with an area under the curve (AUC) of 0.95 for specifically determining Aβ status [13]. A third FTP study found that the application of cut-points in the temporal meta-region of interest (ROI) could separate AD from a group of other neurodegenerative disorders, including Parkinson’s disease (PD) (with or without cognitive impairment), PSP, behavioral variant frontotemporal dementia, dementia with Lewy bodies, CBD, non-fluent variant primary progressive aphasia, semantic variant and primary progressive aphasia, vascular dementia, multiple system atrophy, CTE, and unspecified primary progressive aphasia, with 90.3% accuracy [30]. Other tau PET radiotracers, [^18^F]RO948 and [^18^F]PM-PBB3, have been used for the same purpose. Using [^18^F]RO948 retention in the entorhinal cortex, inferior/middle temporal, fusiform gyrus, parahippocampal cortex, and amygdala, Leuzy et al. was able to differentiate AD from non-AD disorders with an accuracy of 91.3%. The non-AD dementing disorders in their analysis included behavior variant frontotemporal dementia, semantic variant primary progressive aphasia, dementia with Lewy bodies, progressive supranuclear palsy, PD (with or without cognitive impairment), multiple system atrophy, and vascular dementia. Furthermore, the non-AD cases where an appreciable [^18^F]RO948 signal was observed were often Aβ-positive [14]. Using [^18^F]PM-PBB3 retention in the globus pallidus and medial temporal lobe, Endo et al. were able to differentiate AD from PSP via a machine learning-derived neuropathological score [32].

The results from these studies suggest that, with the optimal regional analysis, tau PET can be used to differentially diagnose AD from other neurodegenerative disorders and sources of cognitive impairment. This is important because, at present, the primary differentiator of AD from other sources of cognitive impairment is the presence of Aβ. On the other hand, as discussed later, tau PET has a utility beyond differential diagnosis and can offer a more comprehensive view of AD.

## 3. Tau PET for Staging Disease Progression

**Figure 1:**
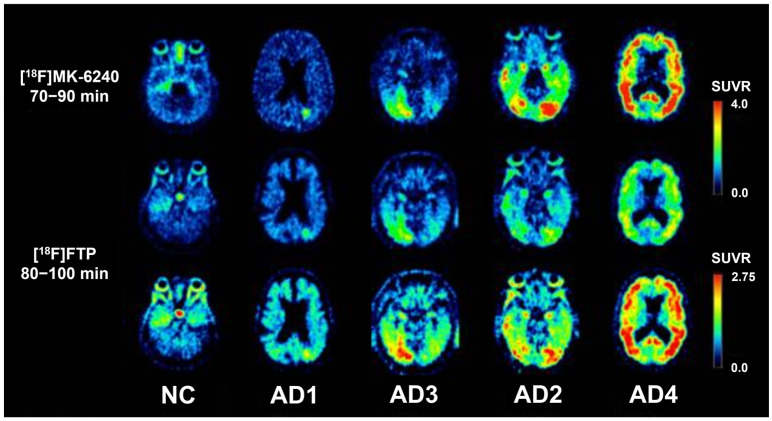
Tau tracers ([^18^F]MK-6240 and [^18^F]FTP), scaled by tracer-specific standardized uptake value ratios (SUVRs), visibly differentiate individuals with low tau and normal cognition (NC) from those with high tau in AD (AD1, AD3, AD2, AD4). This research was originally published in JNM. Alexandra Gogola, Davneet S. Minhas, Victor L. Villemagne, Ann D. Cohen, James M. Mountz, Tharick A. Pascoal, Charles M. Laymon, N. Scott Mason, Milos D. Ikonomovic, Chester A. Mathis, Beth E. Snitz, Oscar L. Lopez, William E. Klunk, and Brian J. Lopresti. Direct Comparison of the Tau PET Tracers 18F-Flortaucipir and 18F-MK-6240 in Human Subjects. J Nucl Med. Jan 2022, 63 (1) 108-116. © SNMMI.

Two characteristics of tau PET position it as a useful tool for staging AD: (1) it closely predicts neurodegeneration and cognitive impairment; (2) it differentiates individuals with AD and MCI due to AD from those with normal cognition, as demonstrated in Figure 1 [33].

Previous studies of tau, including post-mortem and imaging studies, have shown that the density of neurofibrillary tangles (NFTs) and their topographical distribution in the brain is strongly associated with neurodegeneration and cognitive impairment [11,34,35,36,37,38]. Tau tracer retention has a close association with the imaging biomarkers of neurodegeneration, such as MRI-derived cortical gray matter atrophy [16,17,18]. Baseline tau–PET binding has been found to predict the degree and spatial distribution of cortical atrophy at both the group and patient levels [39], with a pronounced association in the mesial temporal lobe (MTL) [40]. These associations are often strongest between tau deposition and future neurodegeneration [39]. The evidence suggests that a higher FTP signal in the inferior temporal region indicates accelerated cortical thinning, possibly representing an inflection point in preclinical AD [41]. Tau tracer retention is also highly predictive of [18F] fluorodeoxyglucose (FDG) PET-determined glucose hypometabolism, reflecting synaptic dysfunction and neuronal loss [39,42,43].

Tau PET imaging has also revealed that, in Aβ+ individuals, increasing levels of cortical tau were associated with increasing impairment in several cognitive domains [44,45,46]. One study of Aβ+ participants found that greater FTP was associated with the metrics of learning, memory, fluency, naming, visuospatial, executive function, and processing speed in the associated regions [47]. Another classified their Aβ+ participants into two groups, those with preclinical AD and those with prodromal AD or dementia. In the preclinical AD group, they found associations between bilateral temporoparietal FTP and global cognition, episodic memory, and processing speed/attention. In the prodromal AD and AD dementia group, they again found associations between bilateral temporoparietal FTP and global cognition, episodic memory, processing speed/attention with additional associations with language and semantic memory [48]. Tau PET’s associations with cognition are not limited to Aβ+ individuals. Work has shown that, in cognitively impaired participants, FTP is directly correlated with language, executive function, and visuospatial performance in the regions localizing with established brain–behavior relationships [49], and, in non-demented participants, RO948-detected tau in the Brodmann area 35, entorhinal cortex, and anterior hippocampus is associated with Alzheimer’s Disease Assessment Scale-Cognitive (ADAS) delayed memory performance [40]. Additionally, across diagnostic groups, the measures of Genentech Tau Probe 1 (GTP1) PET uptake in whole cortical gray matter and the temporal meta-ROI tau are correlated with global cognition cognitive performance [50], and the FTP in the inferior temporal ROI is associated with both global cognition and memory [51].

Several groups have reported significant differences in tau tracer retention between cognitively normal older adult controls and Aβ+ AD patients [44,51,52,53,54,55], as well as in atypical presentations of AD [56,57,58]. A variety of approaches and radiotracers have been successfully employed in this pursuit. One study found that deep learning can be used in conjunction with FTP PET to grade and differentiate between cognitively unimpaired (CU) and AD, as well as CU and MCI. While the additional clinical data improved the classification, imaging alone was sufficient [59]. Another method used clustering based on the FTP uptake in the entorhinal cortex and cortex to split the study participants into three groups and found that both age and clinical presentation mapped well onto the clusters [60]. The SPARE-Tau index developed by Toledo et al. was able to differentiate between not only Aβ positive and negative CU individuals but between clinical diagnoses of Aβ positive CU, MCI, and demented individuals [61]. Topographic staging with FTP based on four levels of Braak-based regions of interest (ROIs) was able to identify distinct patterns of clinical progression [62]. Non-negative matrix factorization has been shown to identify regions of tau aggregation in FTP images that can then be applied in a data-driven staging that reflects AD severity and gene expression [63]. The SuStaIn model [64], with four stable, distinct spatiotemporal patterns is able to identify groups with unique clinical profiles and longitudinal outcomes [65]. Even the visual assessment of FTP has been supported as a reliable method of determining stages in memory clinic patients, with comparable success to analytic SUVR-based methods [66]. Tau tracers other than FTP have shown utility in AD staging. Multiple studies have made use of PI-2620 in differentiating CU, MCI, and AD individuals [67,68], and another has shown that the application of simple cut-offs can separate AD dementia from CU individuals using FTP, RO948, and MK-6240 [15].

The effect of tau on neurodegeneration, cognitive impairment, and clinical phenotype positions tau PET as a powerful tool for staging AD. With the dynamic range of the tau PET signal, it will be possible to identify more precise stages of AD beyond cognitively unimpaired, MCI, and AD, as proposed in the updated National Institute on Aging and Alzheimer’s Association (NIA-AA) biological staging framework [69]. This will allow for the more granular assessment and prediction of disease progression and clinical trial outcomes.

## 4. Tau PET for Prognosis and Therapeutic Trials

Tau PET’s most significant contribution to improving AD diagnostics and therapeutics may be its usefulness in predicting cognitive decline. A significant body of evidence suggests that a strong tau PET signal in Ab+ individuals predicts cognitive decline in the short term [19,20,21] better than Aβ PET alone [70], with tau deposition being closely related to cognitive changes [43]. These results have been replicated across a variety of studies.

**Figure 2:**
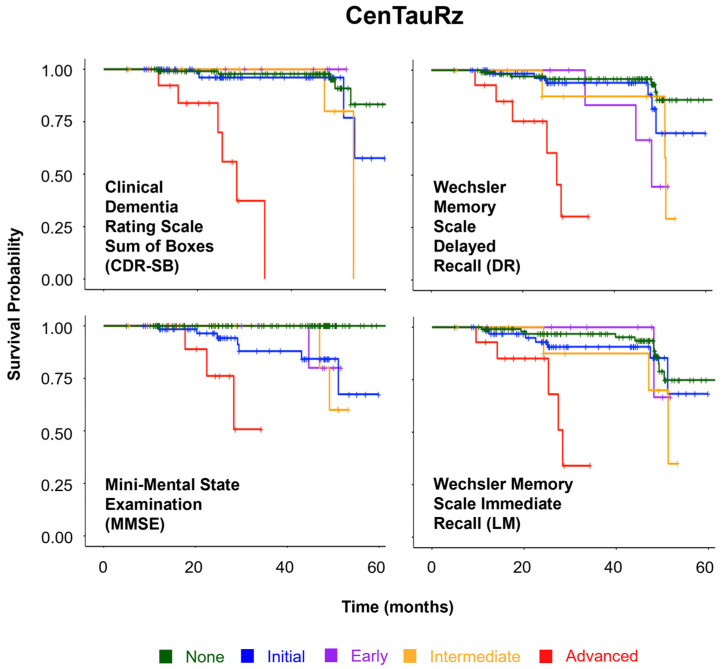
Cognitive decline survival plots for cognitively unimpaired participants’ NIA-AA multilevel tau staging. (DR: Delayed Recall. LM: Logical Memory. CDR_SB: Clinical Dementia Rating Sum of Boxes. MMSE: Mini-Mental State Examination.)

One study with FTP in cognitively normal older adults found Aβ-dependent associations of regional tau deposition with memory decline. In Aβ+ participants, tau in the anterior-temporal and posterior-medial regions predicted memory decline, whereas, in the entorhinal cortex, it predicted memory decline regardless of Aβ-status. Perhaps of greatest interest was the observation that the best predictor of memory decline was tau in the anterior-temporal ROI [62]. Work done by Biel et al. investigating the application of fMRI-derived meta-analytical cognitive maps to FTP tau PET images found that the assessment of tau deposition in cognitive-domain-specific regions outperforms more conventional tau–PET metrics and, when paired with personalized cognitive measures, is more sensitive to AD-related cognitive decline with respect to episodic memory, language, executive functioning, and visuospatial metrics [71]. A study with GTP1 tau PET found that baseline tau was significantly associated with yearly global cognitive change, as measured by the Mini-Mental State Exam (MMSE), Clinical Dementia Rating Sum of Boxed (CDR-SB), 13-item version of the AD Assessment Scale-Cognitive Subscale (ADAS-Cog13), and the Repeatable Battery for the Assessment of Neuropsychological Status (RBANS) Total Index, independent of cortical volume or Aβ-PET. Furthermore, they discovered that, after classifying their participants as T+/−, T+ participants declined more rapidly than T− participants [19]. Similarly, a study with FTP tau PET found that global tau predicts global cognitive and memory decline, as measured by MMSE, ADAS-Cog13, and ADNI-MEM, within approximately 2 years, and that progressively more advanced Braak-stages were associated with an increasing risk of decline and conversion to MCI or dementia [72]. Another study with FTP found that baseline tau was significantly associated with global cognitive and memory decline and that progressive levels of tau deposition predicted faster rates of decline in individuals with both normal and impaired cognition (Figure 2) [73]. Several studies performed with a variety of tau tracers uncovered associations between baseline tau PET and MMSE. Furthermore, [^18^F]RO948 and temporal ROI-detected tau were associated with MMSE in patients with amnestic MCI or mild dementia [74], an FTP-detected neorcortical tau predicted longitudinal change in MMSE across a spectrum of AD clinical presentations [75]; both FTP and RO948-detected baseline temporal tau were associated with MMSE decline [70], and THK5317-detected baseline middle temporal tau strongly predicted change in MMSE [76]. Other evidence of decline goes beyond simple cognitive measures and shows that, in MCI patients, baseline temporal meta-ROI-measured RO948 and FTP predict conversion to dementia [77], and, in AD patients, baseline FTP in transentorhinal, limbic, and isocortical ROIs predict deterioration to psychosis [78].

While tau imaging has already been used in tau therapeutic trials [79], it has been mostly used in anti-Aβ trials [80,81,82,83], where some anti-Aβ monoclonal antibodies actually show a decrease in the tau PET signal [80], while others show an expected slow-down of tau accumulation in the brain [82,83]. These findings suggest tau PET has utility identifying the individuals most likely to benefit from disease-modifying interventions, particularly once the temporal relationships between tau, neurodegeneration, and cognitive decline can be worked out further. Additionally, if tau is proven to have a causal relationship with neurodegeneration and cognitive decline, the reduction of the tau signal in clinical trials may serve as a surrogate endpoint for halting or preventing the progression of cognitive decline.

## 5. Limitations

**Figure 3:**
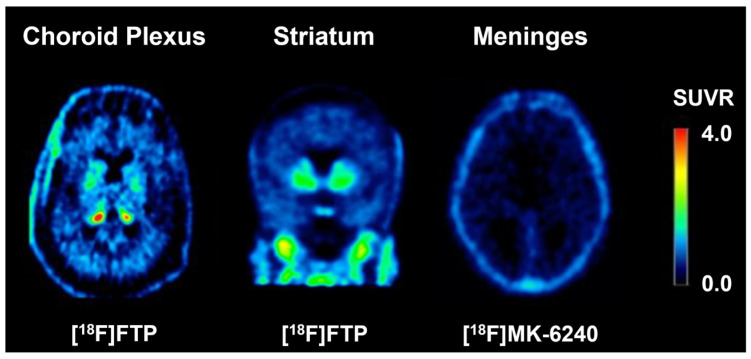
Characteristic tau off-target binding in the choroid plexus, striatum, and meninges. This research was originally published in JNM. Alexandra Gogola, Davneet S. Minhas, Victor L. Villemagne, Ann D. Cohen, James M. Mountz, Tharick A. Pascoal, Charles M. Laymon, N. Scott Mason, Milos D. Ikonomovic, Chester A. Mathis, Beth E. Snitz, Oscar L. Lopez, William E. Klunk, and Brian J. Lopresti. Direct Comparison of the Tau PET Tracers 18F-Flortaucipir and 18F-MK-6240 in Human Subjects. J Nucl Med. Jan 2022, 63 (1) 108-116. © SNMMI.

Although there has been much work to support the use of tau PET as the primary biomarker for AD, it still has several limitations that will need to be addressed before it becomes the standard method of diagnosis and evaluation. As shown in Figure 3 [33], tau PET tracers commonly have areas of off-target retention in locations that can compromise tau signal analysis, including the choroid plexus, basal ganglia, longitudinal sinuses, and meninges [84,85]. This off-target retention is highly variable across tracers and has been shown to be related to other biological variables, including age, sex, and race [86,87,88,89,90]. Additionally, some tau tracers show high levels of non-specific binding, which may inhibit the detection of low levels of tau deposition and explain why they often perform better when differentiating AD from disorders other than MCI [91]. While most AD patients present with both high Aβ and high tau PET levels, approximately 15–20% of subjects diagnosed as probable AD have subthreshold levels of neocortical tau tracer retention [4,5,6]. This may be a consequence of the differential regional onset of Aβ (neocortex) and tau (entorhinal cortex/hippocampus) pathologies, as defined by Thal phases [92] and Braak stages [10]. It may also be tied to the limitations of the currently available tracers (binding affinity, isoform selectivity, tracer kinetics), low binding site concentrations, a low affinity for pretangle pathology, or a problem derived from the thresholds used to determine high and low tau [93].

## 6. Future Directions

While a significant amount of work has been done in recent years to explore the role of tau in the natural history of AD, there is still much left to be done, especially as anti-tau therapies are being developed as potential treatment options. Continued work is needed to develop a methodology for standardizing tau PET outcomes akin to Centiloids for Aβ [94]. There are presently multiple tau radiotracers in use, and studies have compared various tau radiotracers and their respective positivity thresholds [33,95]. One method of standardization that has been proposed, the CenTauR scale, shows great promise but has yet to reach widespread utilization [96]. More longitudinal studies are needed to better understand the role tau deposition plays in promoting neurodegeneration and to evaluate the extent to which existing thresholds are able to predict future cognitive decline, what the magnitude of the decline will be, and when the cognitive decline will present. Furthermore, more work is needed to investigate the mechanisms of tau interaction with other biomarkers of AD neuropathologic change, both specific and non-specific, and non-AD copathologies in disease development/acceleration so that we can better identify what combination of factors need to be treated [69]. This will be especially important as tau PET will act as a biomarker in anti-tau trials, truly testing the association between tau and cognition and revealing other moderating factors. Another area of future investigation is the potential association between tau PET and subjective cognitive complaints. This could very well demonstrate that even more granular divisions of tau positivity are needed to reveal when individuals without objective impairments are experiencing very early subjective cognitive complaints and to ascertain if these are related to tau deposits. Finally, the continued exploration of tau PET in more diverse populations that represent the full range of racialized groups, socioeconomic statuses, and complex demographics affected by AD is necessary. These explorations will provide a more complete picture of the factors influencing the association between tau and both neurodegeneration and cognitive decline.
